# De-Airing Maneuvers After Minimally Invasive and Robotic-Assisted Intracardiac Procedures

**DOI:** 10.21470/1678-9741-2022-0214

**Published:** 2023

**Authors:** Andrea Amabile, Arnar Geirsson, Markus Krane, Gianluca Torregrossa, Tommaso Hinna Danesi, Husam H Balkhy, Theo Kofidis

**Affiliations:** 1 Division of Cardiac Surgery, Department of Surgery, Yale School of Medicine, New Haven, Connecticut, United States of America; 2 Department of Cardiothoracic Surgery, Lankenau Heart Institute, Lankenau Medical Center, Main Line Health, Wynnewood, Pennsylvania, United States of America; 3 Division of Cardiac Surgery, University of Cincinnati College of Medicine, Cincinnati, Ohio, United States of America; 4 Division of Minimally Invasive and Robotic Cardiac Surgery, Department of Surgery, University of Chicago Medicine, Chicago, Illinois, United States of America; 5 Department of Cardiac, Thoracic and Vascular Surgery, National University Heart Centre, Singapore

**Keywords:** Cardiac Surgery, Robotics, Technique, Minimally Invasive, Robotic-Assisted

## Abstract

In the setting of minimally invasive and robotic-assisted intracardiac procedures, de-airing requires further technical considerations due to limited access to the pericardial space and the subsequent difficulty of directly manipulating the heart. We summarize the technical steps for de-airing according to different cannulation strategies for minimally invasive and robotic-assisted intracardiac procedures.

## INTRODUCTION

The completion of intracardiac procedures requires careful de-airing of the left-heart chambers in order to prevent systemic air embolism from occurring, which can lead to severe neurologic and myocardial dysfunction^[1]^. The source of air emboli during restoration of cardiac contractility is multifactorial. Bubbles can originate from (1) air previously accumulated in local recesses of the heart chambers, (2) air entrained through the atriotomy or along the left ventricular (LV) vent from the pericardial space during weaning from cardiopulmonary bypass, or (3) intracardiac cavitation caused by augmented pressure, temperature, and turbulence (particularly due to vigorous shaking of the heart), progressively forcing dissolved carbon dioxide out of the solution into microbubbles which flock and tend to merge into bigger ones (*i.e.*, the “Coca-Cola” effect).

De-airing during minimally invasive and robotic-assisted intracardiac procedures poses additional challenges due to the limited access to the pericardial space and the subsequent difficulty of directly manipulating the heart or placing a needle into the apex of the left ventricle. Additionally, minimally invasive procedures are performed with different cannulation and myocardial protection strategies, each of which entails some differences in the way de-airing is accomplished.

## TECHNIQUE

In minimally invasive intracardiac procedures, possible cannulation strategies are: (1) arrested heart with endothoracic mechanical aortic cross-clamping and antegrade cardioplegia delivered through a cannula placed in the ascending aorta; (2) arrested heart with antegrade cardioplegia delivered through an aortic endoballoon (Intraclude^TM^ Edwards, Irvine, California, United States of America); and (3) fibrillating heart with cardiopulmonary bypass support. Regardless of the cannulation strategy, continuous CO₂ insufflation of the chest cavity (the rationale for which is based on its favorable coefficient of solubility^[2]^) and Trendelenburg positioning of the patient when possible (which aids in directing any bubbles towards the aortic root and the LV apex) should be used.

**Fig. 1:**
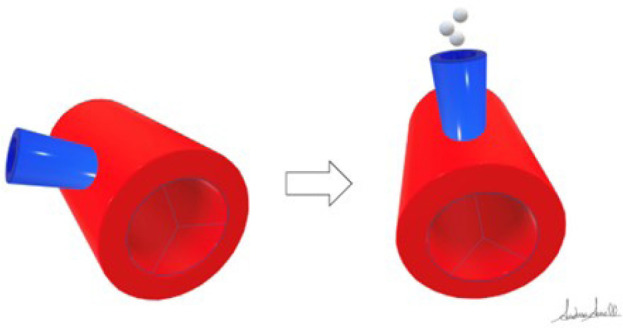
Schematization of the ascending aorta (red) and the aortic cardioplegia cannula (blue) from a caudo-cranial perspective. In normal positioning (left), the cannula enters the ascending aorta with an acute angle. After rotating the patient to his left side (right), the cardioplegia cannula becomes perpendicular to the horizontal plane and thus facilitates the outflow of air bubbles.

In the first scenario, de-airing is carried out in three steps which are similar to the ones routinely performed in open procedures as described by Carpentier et al.^[3]^. Passive retrograde de-airing is first performed with the endothoracic aortic cross-clamp in place and with the cardioplegia suction line off. The atrial suture line is loosened while the heart is partially filled by reducing the venous return from the cardiopulmonary bypass machine and the left lung is inflated in order to roughly mobilize air collected in the pulmonary veins. Then, the first suture line of the left atriotomy is tied and suction on the aortic cardioplegia line (aortic root vent) is initiated while the endothoracic aortic cross-clamp is still in place and the heart is mildly filled. This passive antegrade de-airing allows to evacuate most of the remaining air from the heart cavities through the cardioplegia line without risk for systemic embolism. Differently than in median sternotomy, in minimally invasive procedures, the cardioplegia cannula does not enter the proximal ascending aorta with an orthogonal angle to the horizontal plane but forms an acute angle instead, although the acuteness of this angle can vary greatly. Thus, it can be beneficial to tilt the operating table approximately 20 degrees towards the patient’s left side in order to move the cardioplegia cannula orthogonally to the horizontal plane and thus facilitate the outflow of air bubbles (Figure 1). This strategy is obviously not possible in robotic procedures as the table is fixed in position while the robot is docked, and in such cases, a percutaneous antegrade catheter inserted laterally to the right internal thoracic artery can be close to vertical. Finally, the endothoracic clamp is released while maintaining the cardioplegia suction line on heavy suction (active antegrade de-airing) and the patient in Trendelenburg position (in non-robotic cases) in order to minimize the risk of cerebral embolism, and the second layer of the atriotomy is closed. Continuous monitoring through intraoperative transesophageal echocardiography (TEE) is mandatory to detect any residual air in the left cavities, with particular attention for air pockets that may collect along the papillary muscles, the interventricular septum, and the apex, and in the aortic root. When the patient is completely weaned from cardiopulmonary bypass and no residual air is detected, the cardioplegia line is removed. In robotic and totally endoscopic cases, an LV vent across the mitral valve (MV) is usually necessary as the root vent is usually removed and the site repaired safely prior to unclamping.

In the second scenario, the endoballoon works as intra-aortic occlusion device, aortic root vent, and antegrade cardioplegia line at the same time, due to its triple-lumen structure. In this setting, an additional vent should be placed through the MV in order to provide enhanced de-airing. This can be safely removed just before closure of the left atriotomy, with completion of the de-airing by the endoballoon suction line (root vent). Similar to the first scenario, the endoballoon can be then removed when the patient is completely weaned from cardiopulmonary bypass and no residual air is detected on intraoperative TEE.

In the third scenario, complete de-airing must be achieved before cessation of ventricular fibrillation (VF) and restoration of ventricular ejection. During VF arrest, an LV vent must be placed through the MV continuously throughout the procedure. Once the repair is completed, the vent is gently advanced to the apex of the left ventricle under vision in order to maximize the likelihood of suctioning air bubbles. The vent must be set on high suction while slowly filling the heart and insufflating the left lung. Careful avoidance of ventricular distention is important to prevent increased oxygen demand. Once air evacuation is confirmed by TEE, the LV vent is removed, and the left atrial suture line closure is completed. In case sinus rhythm is spontaneously regained during de-airing, the MV must be kept incompetent to prevent active ejection and subsequent air embolism.

Coronary air embolism must be suspected in the incident of reduced cardiac contractility, acute changes in the electrocardiogram suggestive of ischemia, or if frequent extrasystoles or ventricular tachycardia are noticed in the absence of other possible etiologies. If this scenario is observed after removal of all venting lines, cardiopulmonary bypass assistance must be sustained for an extra amount of time with a targeted perfusion pressure between 70 and 80 mmHg while gently filling the heart. This is usually enough time for coronary air emboli to be washed through spontaneously. Small ejections are allowed, but non encouraged. The role of increasing ventricular rate by pacing to move trapped air is controversial, as this may also exaggerate ventricular strain and increase oxygen demand. If these actions do not relieve the situation and motion abnormalities persist, one must immediately suspect coronary artery compromise and escalate accordingly.

## DISCUSSION

De-airing following intracardiac procedures must be meticulously performed in order to avoid systemic air embolism. Vigorous and continuous shaking of the heart should be used judiciously (to break up large pockets of air when present), if at all, otherwise the opposite effect ensues, namely enhanced bubbles production. Additionally, direct manipulation of the heart may be challenging during minimally invasive and robotic-assisted procedures because of the limited access to the pericardial space and the heart. In such circumstances, specific technical precautions must be taken according to the strategy of choice for cannulation and aortic occlusion.

Conditions specific to the robotic, totally endoscopic approach are:

1. Table maneuvers are not possible given the obligatory fixed table position while the robot is docked.
2. The dynamic atrial retractor serves as a nice tool to gently elevate the heart by lifting up the inferior wall and apex after closure of the left atrium and filling of the heart.
3. The antegrade catheter is usually removed and the aortic insertion site repaired prior to release of the endothoracic clamp in totally endoscopic procedures. Therefore, maintaining a vent across the MV is necessary to continue to de-air after the clamp is removed.

## CONCLUSION

**Fig. 2:**
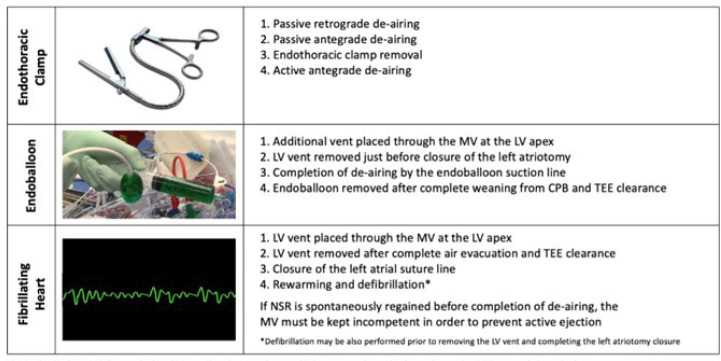
De-airing steps according to different cannulation strategies. CPB=cardiopulmonary bypass; LV=left ventricular; MV=mitral valve; NSR=normal sinus rhythm; TEE=transesophageal echocardiography.

In conclusion, de-airing during minimally invasive and robotic-assisted intracardiac procedures may entail some additional technical challenges. Various techniques are available (Figure 2) to accomplish proper de-airing safely and effectively in this setting.
